# A novel signature for predicting prognosis and immune landscape in cutaneous melanoma based on anoikis-related long non-coding RNAs

**DOI:** 10.1038/s41598-023-39837-5

**Published:** 2023-09-28

**Authors:** Miao Zhang, Yuzhi Zuo, Jian Guo, Lushan Yang, Yizhi Wang, Meiyun Tan, Xing Guo

**Affiliations:** 1 Department of Plastic and Burns Surgery, Affiliated Hospital of Southwest Medical University, Luzhou, Sichuan China; 2 Vascular Surgery Department, Affiliated Hospital of Southwest Medical University, Luzhou, Sichuan China; 3 Department of Orthopaedics, Affiliated Hospital of Southwest Medical University, Luzhou, Sichuan China; 4 Center of Ambulatory Surgery, Affiliated Hospital of Southwest Medical University, Luzhou, Sichuan China

**Keywords:** Cancer, Cancer genetics, Cancer genomics, Cancer microenvironment, Cancer models, Cancer prevention, Cancer therapy, Skin cancer, Tumour biomarkers, Tumour immunology, Immune cell death, Tumour immunology, Computational biology and bioinformatics, Computational models, Functional clustering, Genome informatics

## Abstract

Anoikis is a unique form of apoptosis associated with vascularization and distant metastasis in cancer. Eliminating anoikis resistance in tumor cells could be a promising target for improving the prognosis of terminal cancer patients. However, current studies have not elaborated on the prognosis effect of anoikis-related long non-coding RNAs (lncRNAs) in cutaneous melanoma. Pre-processed data, including RNA sequences and clinical information, were retrieved from TCGA and GTEx databases. After a series of statistical analyses, anoikis-related lncRNAs with prognostic significance were identified, and a unique risk signature was constructed. Risk scores were further analyzed in relation to the tumor microenvironment, tumor immune dysfunction and exclusion, immune checkpoint genes, and RNA methylation genes. The indicators were also used to predict the potentially sensitive anti-cancer drugs. An anoikis-related lncRNAs risk signature consisting of LINC01711, POLH-AS1, MIR205HG, and LINC02416 was successfully established in cutaneous melanoma. Overall survival and progression-free survival of patients were strongly linked with the risk score, independently of other clinical factors. The low-risk group exhibited a more beneficial immunological profile, was less affected by RNA methylation, and was more sensitive to the majority of anti-cancer drugs, all of which indicated a better prognostic outcome. The 4 hub lncRNAs may be fundamental to studying the mechanism of anoikis in cutaneous melanoma and provide personalized therapy for salvaging drug resistance.

## Introduction

Cutaneous melanoma is a form of skin cancer that develops from the malignant proliferation of melanocytes, with poor detection in early stages, prone to metastasis, and higher mortality^1^. However, the etiology of melanoma is not entirely clear. Potential carcinogenic factors include overexposure to ultraviolet light and the consequent sunburn of the skin, medical history of dysplastic nevi, multiple nevi and family history of melanoma, and immune system deficiency^2,3^. Based on global cancer incidence statistics for the year 2020, a research team predicted a 50% improvement in the incidence of melanoma patients and a 68% increase in mortality by 2040^4,5^. With the utilization of three categories of anti-cancer drugs, namely BRAF-targeted, MEK-targeted, and Immune checkpoint inhibitors (ICIs) on melanoma, significantly improved OS had been achieved in advanced-stage patients^6–8^. However, the widespread increase in the occurrence of drug resistance and adverse reactions gradually aggravates the possibility of poor treatment outcomes^9–11^. Therefore, the discovery of new biomarkers could assist in diagnosis, decrease drug resistance, inhibit tumor progression, and improve survival prognosis.

When cells detach from the extracellular matrix (ECM) or adherent cells, both internal and external pathway signaling pathways are activated to modulate mitochondrial function, which normally induces a particular form of programmed cell death to maintain cellular homeostasis called anoikis^12^. However, suppression of anoikis could activate signaling pathways, including TGF-β, TrkB, and PI3K/AKT, which induce epithelial–mesenchymal transition, leading to tumor cell proliferation and metastasis^13,14^. Inducing anoikis in melanoma cells would not only inhibit remote metastasis but also would enhance sensitivity to chemotherapeutic drugs, which offers a novel bio-pathway for cancer-targeted therapies^15^. Yet, more research is needed to explore the effect of anoikis on anti-tumor immunity.

Long non-coding RNAs (LncRNAs), with sequences of over 200 nucleotides, function to modulate the expression of genes without altering the nucleotide sequences^16^. LncRNAs, including ANRIL, HOTAIR, MALAT1, SAMMSON, etc., have been implicated to play an essential part in tumor progression, metastasis, and anti-tumor immunity in melanoma via numerous research^17,18^. Furthermore, several lncRNAs may modulate immune cell activity in the tumor microenvironment (TME) and induce tumor immune evasion or anti-tumor immunity, which provide potential possibilities for addressing immunotherapy resistance in poor-prognosis cancers^19–21^. Nevertheless, there is still a lack of quantitative evidence to precisely interpret the function of anoikis-related lncRNAs in the treatment resistance and progression of melanoma.

The current study established a clinical prognostic model by filtering out anoikis-related lncRNAs in melanoma and exploring their prognostic value. Furthermore, the interaction among risk score with RNA methylation and immune features revealed that anoikis-related lncRNAs could be potential biomarkers to alleviate resistance to targeted and immunotherapy drugs, providing adjuvant therapy to improve the prognosis of late-stage melanoma patients. Our research establishes a viable theoretical foundation for understanding the precise mechanism underlying the function of anoikis-related lncRNAs in melanoma.

## Materials and methods

### Data capture and pre-processing

We extracted RNA sequences and clinical statistics from the TCGA database for 471 cutaneous melanoma samples and 1 normal tissue sample, and we collected RNA expression data from the GTEx database for 812 normal skin tissues. Afterward, all RNA data were combined and normalized using R and Perl programming languages.

### Identification of anoikis-related lncRNAs

A number of 67 anoikis-related genes (ARGs), with a relevance score of no less than 2.0, were retrieved from the GeneCards database (https://www.genecards.org) (Supplementary Table I). We examined the RNA expression data of all samples using Pearson correlation analysis to detect lncRNAs linked with anoikis genes based on the criterion of |correlation coefficient| (|CC|) > 0.4 and P < 0.001. Furthermore, differential analyses were also conducted to identify anoikis-related lncRNAs depending on the standards of |log2 fold change| (|logFC|) > 2 and false discovery rate (FDR) < 0.05.

### Construction of the anoikis-related lncRNAs' prognostic model

To construct a model with clinical predictive value, anoikis-related lncRNAs in SKCM samples were evaluated employing univariate Cox (uni-Cox) regression, and lncRNAs with P < 0.05 were deemed prognostically significant. Next, all tumor samples were separated into train and test cohorts with a 7:3 ratio. This procedure was repeated 100 times at random to eliminate errors. Using the least absolute shrinkage and selection operator (LASSO) regression analysis, anoikis-related hub lncRNAs were identified when results matched tenfold cross-validation and P < 0.05. A novel formula was eventually generated under the muti-Cox regression, as follows:

$$\mathrm{Risk Score}={\sum }_{i=1}^{n}{\mathrm{\alpha }}^{i}\times {\beta }^{i}$$

where n represents the number of lncRNAs inside the model, ${\alpha }^{i}$ is the expression of lncRNA, and ${\beta }^{i}$ is the coefficient of lncRNA. All SKCM samples inside the training and testing sets were classified into distinct risk groups based on their scores compared to the average value.

### Validation of the capability of the risk model to predict clinical characteristics

Kaplan–Meier (K–M) survival analysis was conducted about age, gender, cancer stage, and progression-free survival (PFS) in the overall cohort. Associations between risk score, gender, age, and cancer stage were examined using uni-cox and muti-cox regression. And based on these results, a nomogram was developed to estimate the probable OS of SKCM patients at 1-, 3-, and 5-year with various clinical characteristics. Moreover, we forecasted the ROC curve area at 1-, 3-, and 5-year and compared the 5-year trends to the ROC curves for age, gender, and stage. Finally, the concordance index of risk signature was also compared with clinical indicators.

### Functional pathway enrichment analysis

Differential analysis was performed to obtain differentially expressed genes (DEGs) between the risk groups. Subsequently, to investigate the functional pathways of these gene enrichments, Gene Ontology (GO) as well as Kyoto Encyclopedia of Genes and Genomes (KEGG) analyses were applied to genes fulfilling the |logFC|> 2 and FDR < 0.05 criteria.

### Evaluation of immune-related features

The risk scores of samples were merged with the immune infiltration files downloaded from the CIBERSORT platform (http://timer.cistrome.org/). Then Spearman Correlation Analysis was used to examine the immune infiltration at a distinct risk level. Furthermore, the tumor microenvironment (TME) scores, tumor immune dysfunction and exclusion (TIDE), and immune checkpoint genes (ICGs) (Supplementary Table II) expression were analyzed between the high- and low-risk group. ICGs were retrieved from GeneCards databases using the keyword of immune checkpoint genes.

### RNA methylation-related gene expression

We collected three types of RNA methylation-related genes, consisting of N6-methyladenosine (m6A), C5-methylcytidine (m5C), and N7-methylguanosine (m7G), from a combination of published studies and GeneCards databases (Supplementary Table III). To study the association between risk signature and RNA methylation, we compared and analyzed the differences in these methylation-related genes across samples from high- and low-risk groups.

### Anti-cancer drug sensitivity forecasting

Relevant drug sensitivity studies based on expression data acquired from the cancer drug sensitivity genomics database, employing the “oncoPredict” R package to assess differences in drug half maximal inhibitory concentration (IC50) between high- and low-risk groups.

### Statistics analysis

**Figure 1:**
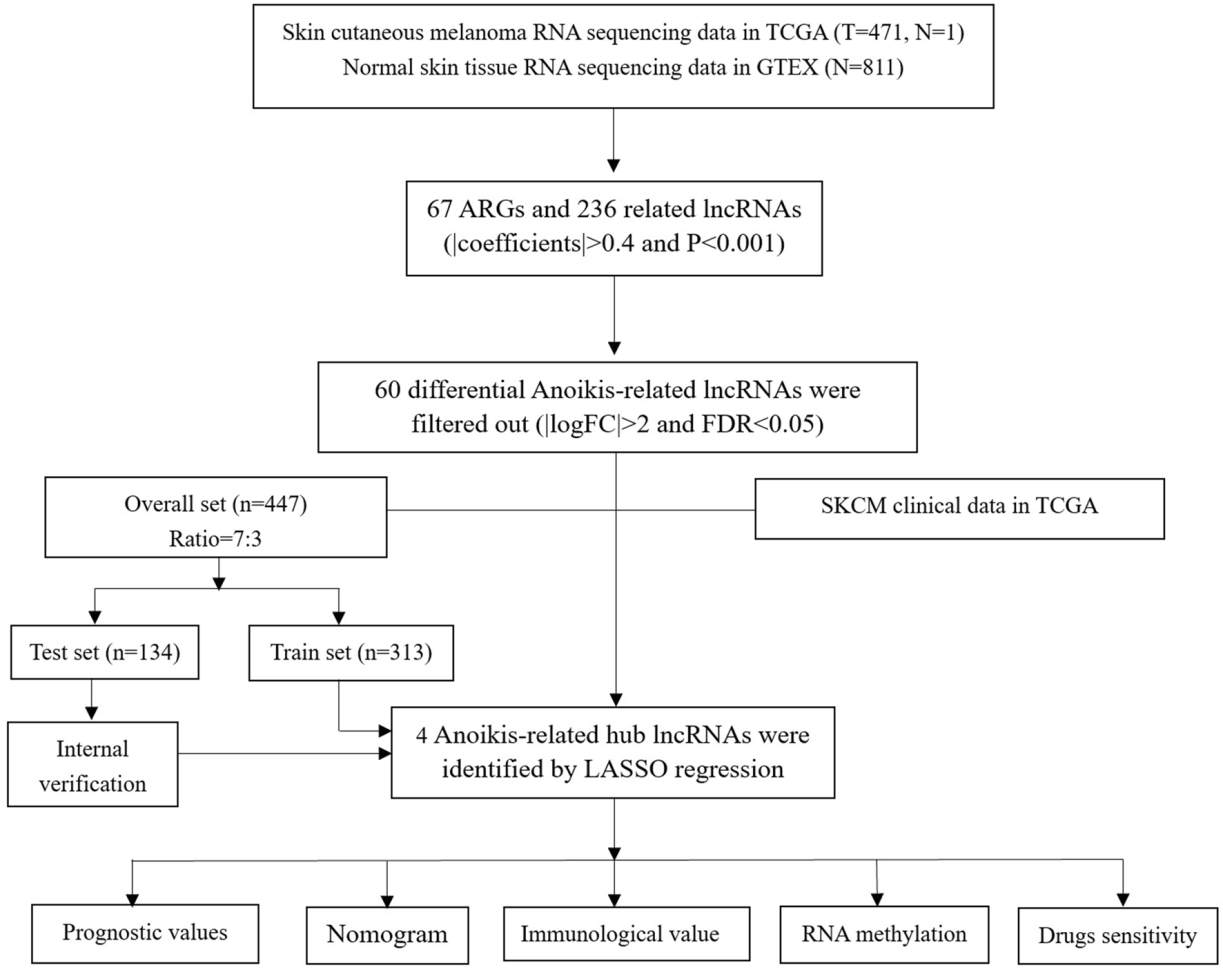
Flow chart of the research.

In the present research, all statistical analyses were undertaken using R statistical programming languages. For the Pearson correlation coefficient test, the criteria were |CC|> 0.4 and P < 0.001. Variance analysis was based on the criteria of |logFC|> 2 and FDR < 0.05. Sensitive anti-cancer drugs were screened at a value of P < 0.001. And other analyses were considered statistically significant at P < 0.05 (***P < 0.001, **P < 0.01, *P < 0.05). Some of the R packages participated in the analysis, including “limma”, “survival”, “caret”, “glmnet”, “survminer”, “timeROC”, “ggplot2”, “galluvial”, “tidyverse”, “ggExtra”, “pheatmap”, “rms”, “pec”, “regplot”, “reshape2”, “ggpubr”, “circize”, “RColorBrewer”, and “oncoPredict”. The flow chart of this study is shown in Fig. 1.

## Results

### Differentially expressed anoikis-related lncRNAs

**Figure 2:**
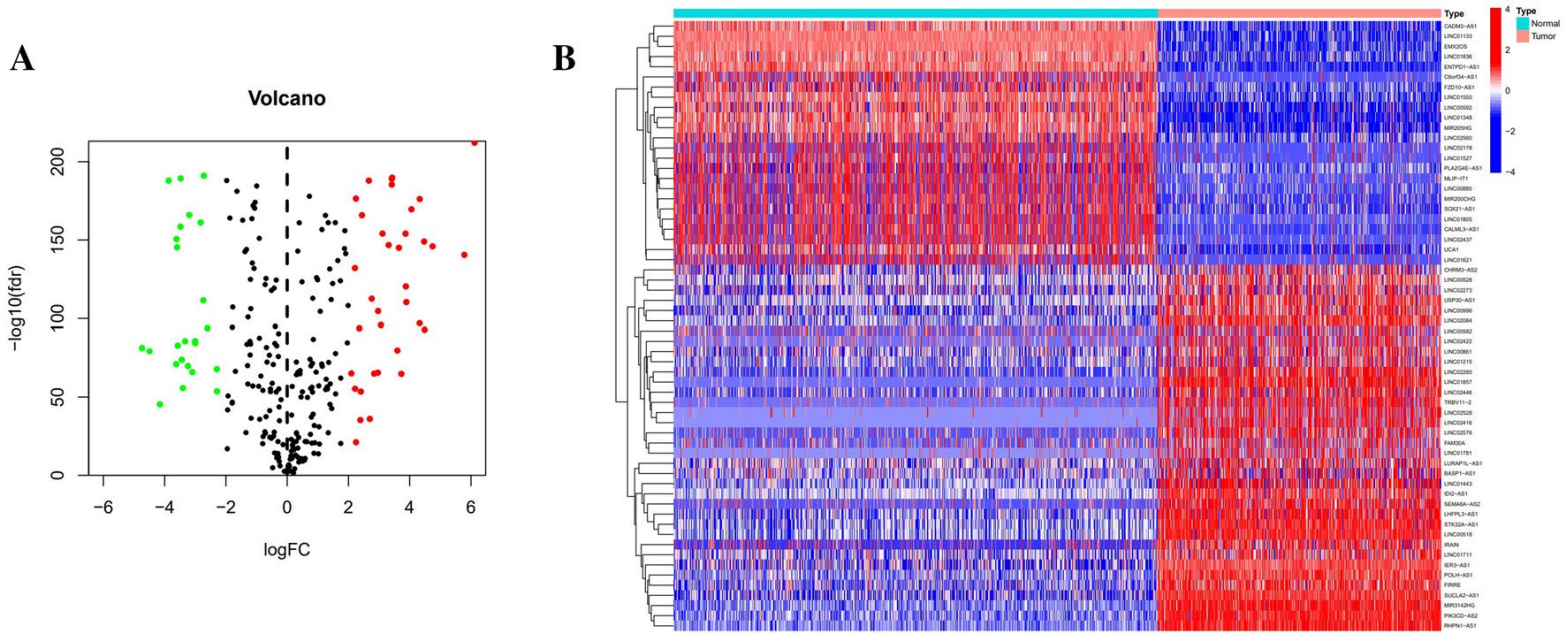
Detection of prominently expressed Anoikis-related lncRNAs in melanoma. (**A**) Volcano diagram of differential lncRNAs with the filtering criteria of |logFC|> 2 and FDR < 0.05. (**B**) Expression heat map of that 60 Anoikis-related lncRNAs in cutaneous melanoma and normal tissues.

A total of 236 lncRNAs were regarded as having linkage to anoikis-related genes by Pearson correlation analysis (|CC|> 0.4 and P < 0.001). Furthermore, by differential analysis, we acquired 60 differential anoikis-related lncRNAs between cancer and normal tissue samples (|logFC|> 2 and FDR < 0.05) (Fig. 2A, Supplementary Table IV), out of which, 24 lncRNAs exhibited hyperexpression in normal samples, whereas the expression of the other 36 lncRNAs was positively correlated with tumors, as presented by visualization of the heat map (Fig. 2B).

### Risk signature development and verification of anoikis-related lncRNAs

**Figure 3:**
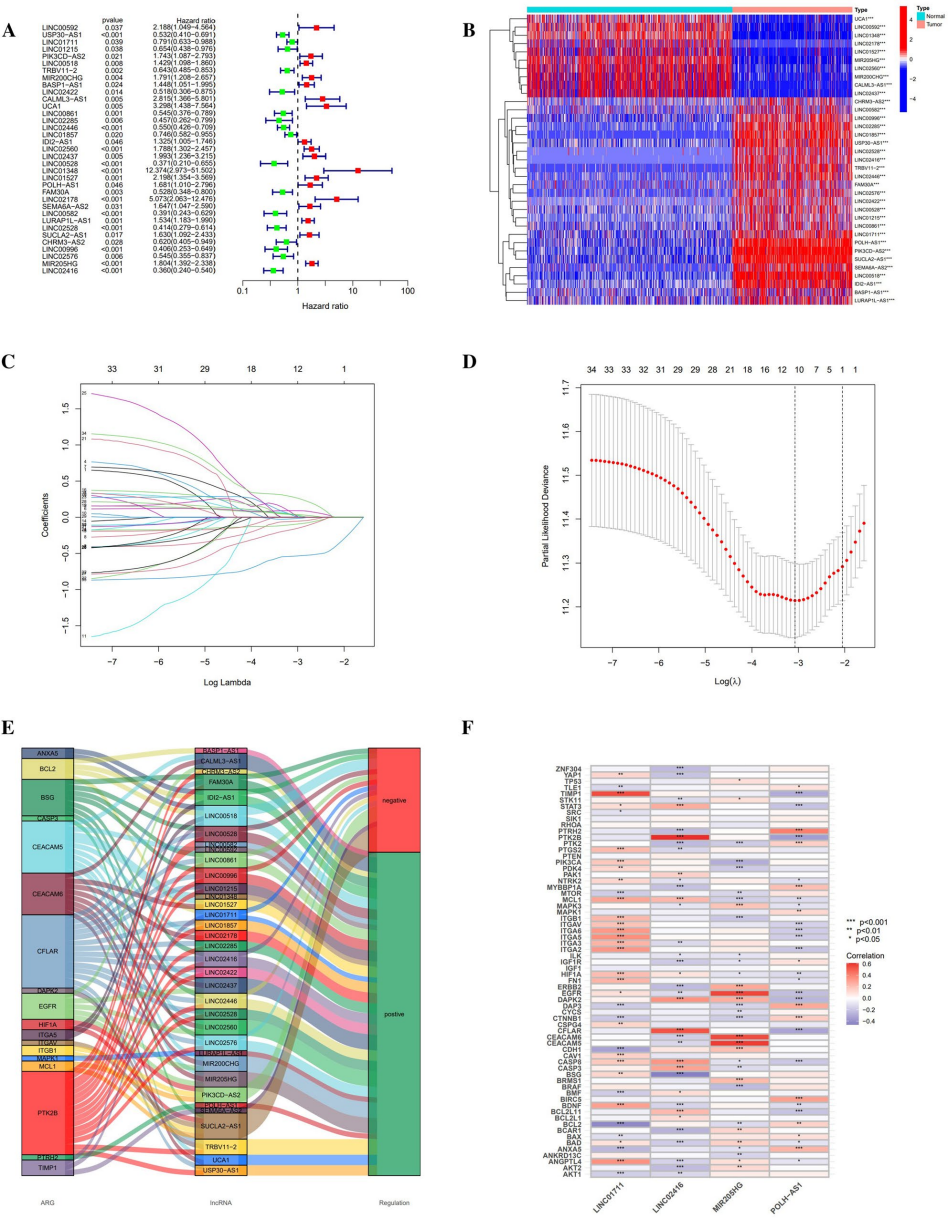
To identify Anoikis-associated lncRNAs in melanoma. (**A**) Forest plot of 35 prognostic-valued lncRNAs with p < 0.05. (**B**) Heat map of prognostic lncRNAs, of which 10 were low- and 25 were high-expressed for tumor tissue. (**C**) Tenfold cross-validation results for the LASSO model. (**D**) Filtering results of the optimum LASSO coefficients for constructing risk signature. (**E**) Sankey plot of the relationship between 35 prognostic lncRNAs and ARGs with coef > 0.4 and P < 0.001. (**F**) Correlation heat map of the 4 hub lncRNAs with ARGs.

**Figure 4:**
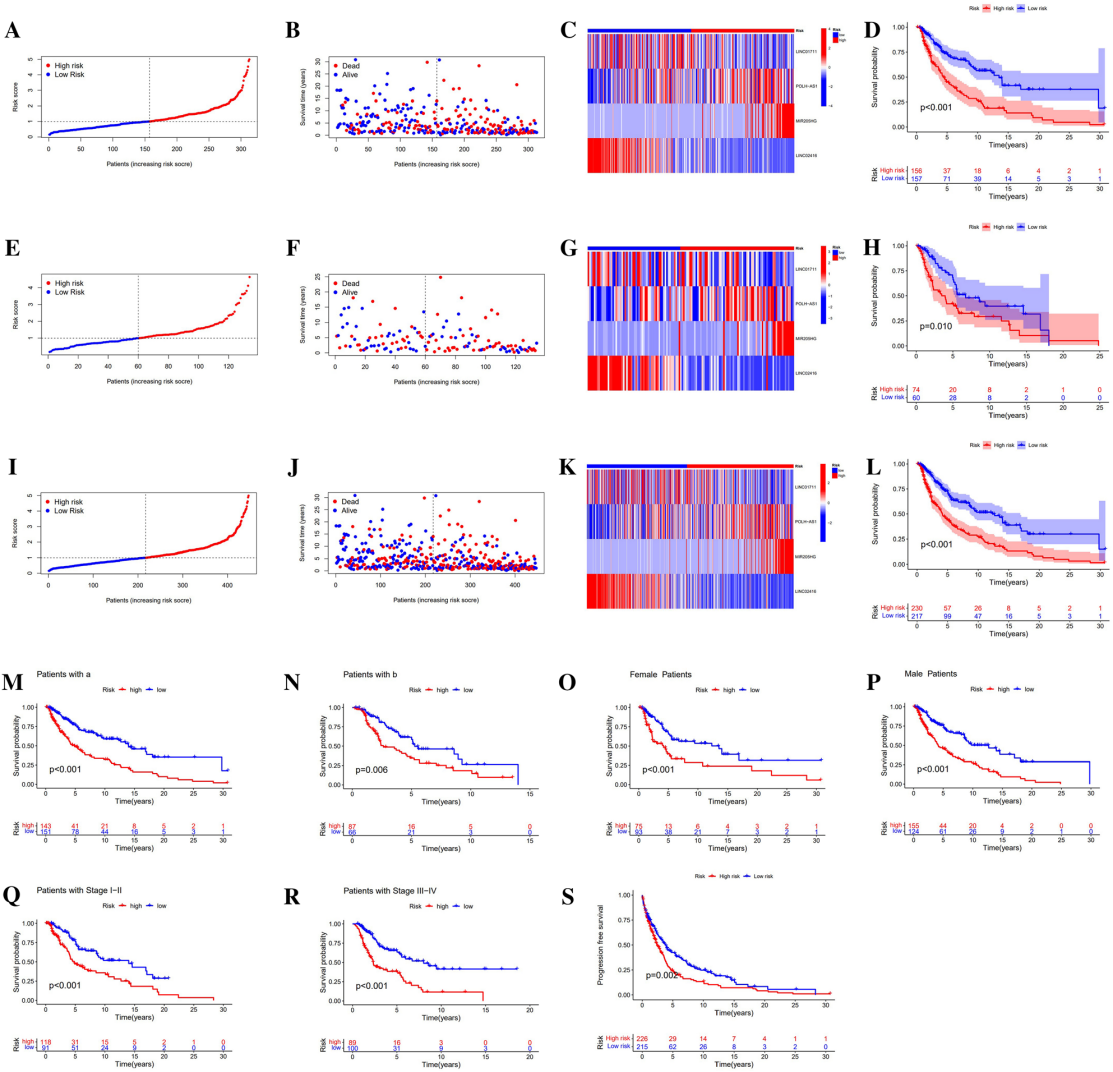
Predictive capability of the risk signature. (**A**–**D**) Risk curve, scatter plot, expression heat map, and K–M plot of the training set. (**E**–**H**) Risk curve, scatter plot, expression heat map, and K–M plot of the test set. (**I**–**L**) Risk curve, scatter plot, expression heat map, and K–M plot of the overall samples. (**M**–**N**) K–M curves of OS in different age levels (a: age ≤ 65, b: age > 65). (**O**–**P**) K–M curves of OS in female and male. (**Q**–**R**) K–M curves of OS in stages I–II and III–IV of melanoma. (**S**) K–M curves of PFS.

Uni-cox regression analysis was performed to know the relationship between risk scores of tumor samples and their survival status information, and 35 differentially expressed anoikis-related lncRNAs were consequently found to be relevant to prognosis (Fig. 3A). Meanwhile, it was recognized from the results of the heat map that 25 anoikis-related prognostic lncRNAs exhibited a trend of highly expressed in the tumor, while another 10 lncRNAs had a negative correlation with the tumor (Fig. 3B). These prognosis-associated lncRNAs were then chosen using the LASSO regression analysis, with a total of 12 remarkable lncRNAs extracted when they conformed to cross-validation with minimal error (Fig. 3C,D). Furthermore, 4 anoikis-related hub lncRNAs, LINC01711, POLH-AS1, MIR205HG, and LINC02416, were located using the muti-cox regression and applied to establish the risk signature. Consequently, that risk signature score would be calculated using the following formula: LINC01711 × (− 0.2956) + POLH-AS1 × (0.4397) + MIR205HG × (0.4625) + LINC02416 × (− 0.9641). Besides, we not only demonstrated the correspondence between 35 prognosis-associated lncRNAs and anoikis-related genes through Sankey diagrams (|CC|> 0.4 and P < 0.001) (Fig. 3E) but also exemplified the degree of correlation between 4 hub lncRNAs and anoikis-related genes using correlation heat maps (Fig. 3F). Consistent results were shown for the risk curves, scatter plots, expression heatmap, and K–M curves plots in the training set, test set, and overall sample (Fig. 4A–L). K–M curves according to age segmentation, gender, and cancer stage were also obtained with significant differences in OS across the high- and low-risk groups (P < 0.05) (Fig. 4M–R). In addition, the K–M curve for PFS in the low-risk group was superior to those in the high-risk group (Fig. 4S). Not only did the above results prove a better prognosis for low-risk score based on the risk signature of anoikis-related lncRNAs, but they also revealed the uniqueness and dependability of this risk signature.

### Nomogram establishment and verification

**Figure 5:**
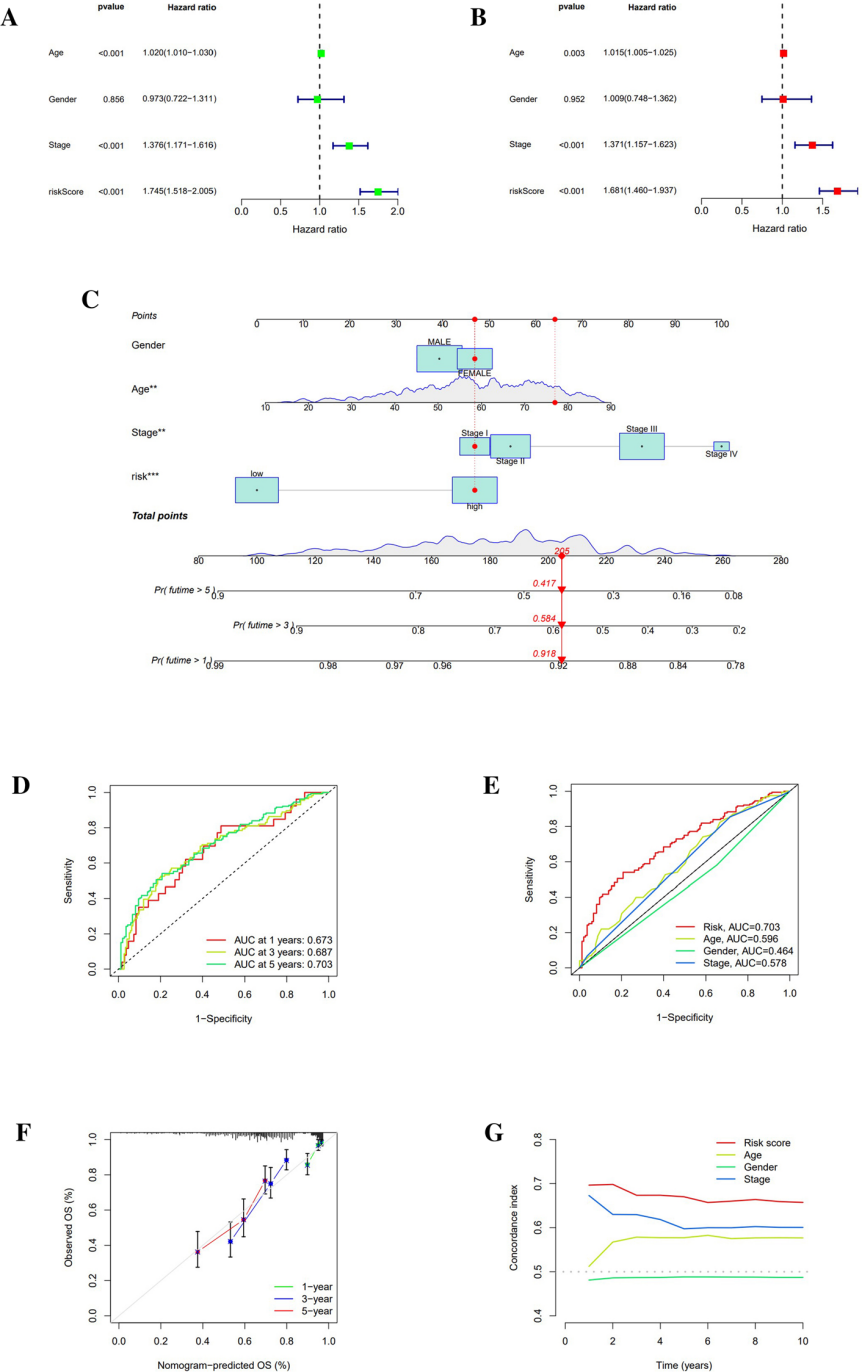
To construct a novel predictive signature. (**A**,**B**) Forest plots of uni-Cox and muti-Cox for risk signature compared to age, gender, and cancer stage. (**C**) Nomogram for risk signature. (**D**) The 1-, 3-, and 5-year ROC curve area ratios were correspondingly 0.673, 0.687, and 0.703. (**E**) The ROC curve area ratios for risk score, age, gender, and stage were correspondingly 0.703, 0.596, 0.464, and 0.578. (**F**) Calibration curves for 1-, 3-, and 5-year OS demonstrated the high predictive accuracy of Nomogram. (**G**) Concordance index of risk scores in comparison to age, gender, and cancer stage.

Both risk signature and cancer stage were significantly affiliated with OS, as demonstrated by forest plots of either uni-Cox regression or muti-Cox regression. And the risk signature was compared with age, gender, and cancer stage, which could be a meaningful independent predictor (Fig. 5A,B). Therefore, we constructed a nomogram based on age, gender, cancer stage, and risk score to predict the odds of survival for SKCM patients at 1-, 3-, and 5-year forward (Fig. 5C). The ROC curve areas for 1-, 3-, and 5-year corresponded to 0.673, 0.687, and 0.703, which were statistically higher than the ROC curve areas for age, gender, and stage (Fig. 5D,E). Not only did the calibration plots show that the nomogram accurately predicted OS for patients at 1-, 3-, and 5-year (Fig. 5F), but also the concordance index of risk score was superior to stage, age, and gender in that order (Fig. 5G).

### GO and KEGG enriched functional pathways

**Figure 6:**
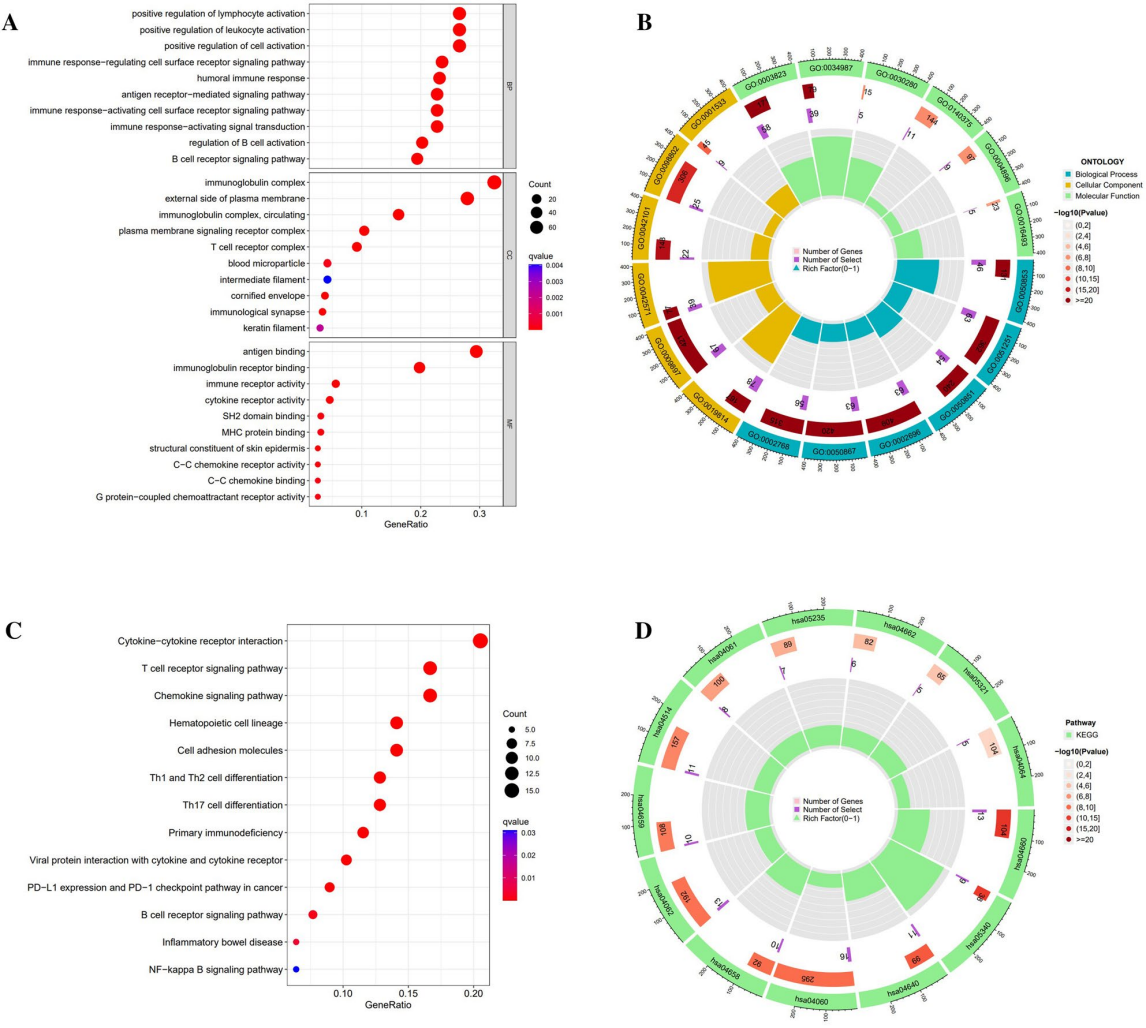
GO and KEEG enrichment pathways for melanoma in distinct risk scores. (**A**,**B**) GO analysis enriched items and corresponding cluster circle diagram. (**C**,**D**) KEEG analysis enriched items and corresponding cluster circle diagram.

By GO analysis, DEGs between risk groups were mainly enriched in functional pathways associated with immunity, plasma membrane, cytokine receptors, and others. In particular, the activation of B cells, lymphocytes, leukocytes, and multiple immune response signal pathways (Fig. 6A,B). Referring to the literatures about the KEGG database^22,23^, we analyzed the functional pathway enrichment of the risk signature. The results of KEGG analysis showed that DEGs were significantly enriched in many functional pathways, including cytokine-cytokine receptor interaction, T cell receptor, chemokine receptor, B cell receptor, cell adhesion molecules, Th1, Th2, Th17 cell differentiation, PD-L1 expression, and PD-1 checkpoint, and hematopoietic cell lineage (Fig. 6C,D).

### Association with immune infiltration, TME, ICGs, and TIDE

**Figure 7:**
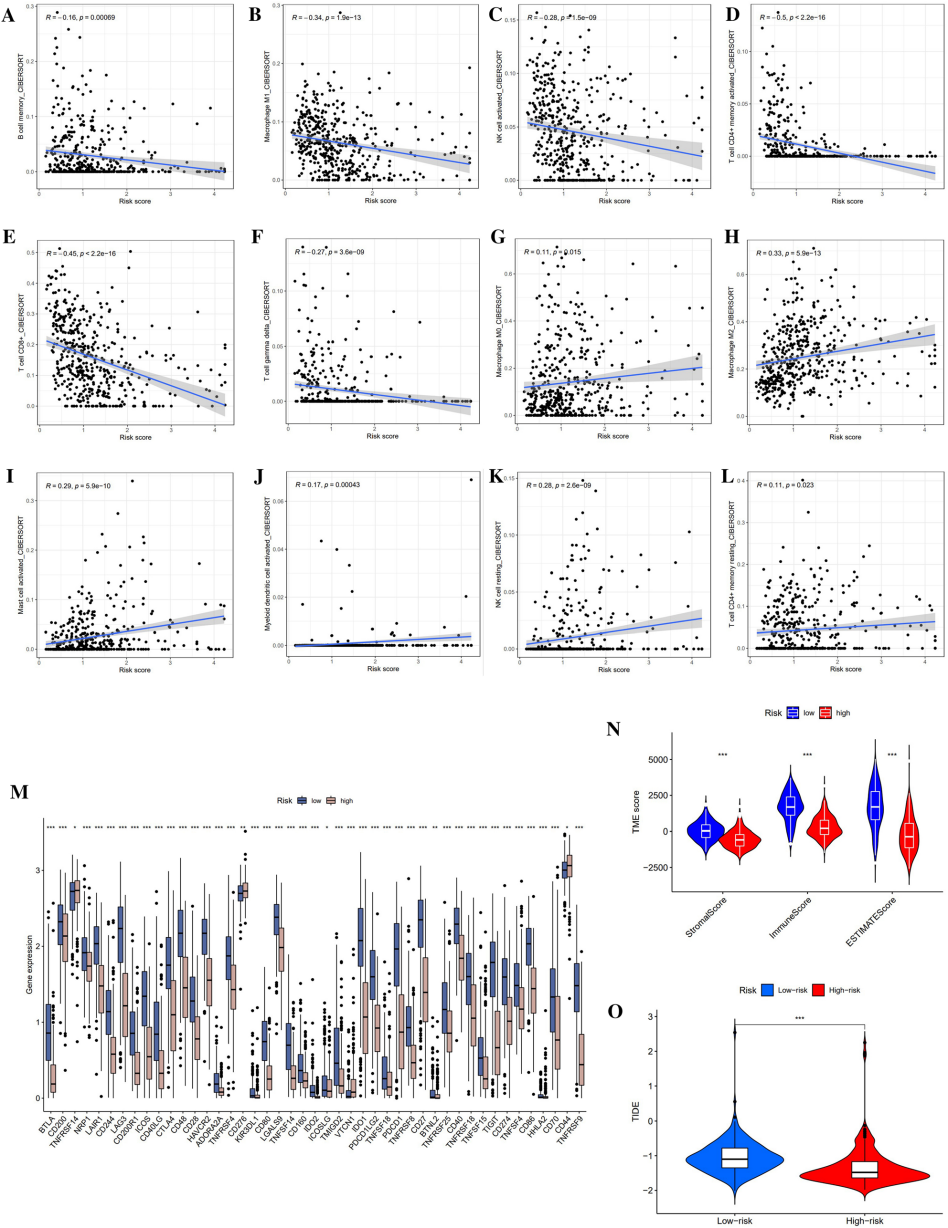
Relationship between risk signature and immunotherapy. (**A**–**L**) Immune infiltration prediction from the CIBERSORT platform. (**M**) Immune checkpoint gene expression in distinct risk scores. (**N**) TME (including stromal, immune and ESTIMATE) score comparisons between risk groups. (**O**) TIDE comparison between risk groups.

From the GO and KEGG pathway analysis results, we learned that anoikis could be specifically related to TME. Therefore, we carved out diverse immunological profiles between high- and low-risk groups. For the predicted outcomes of immune infiltration based on the CIBERSORT platform, B cell memory, Macrophage M1, NK cell activated, T cell memory activated, T cell CD8+, and T cell gamma delta were negatively associated with risk score (P < 0.05) (Fig. 7A–F). In contrast, as the risk score increases, the cells ratio of Macrophage M0, Macrophage M2, Mast cell activated, Myeloid dendritic cell activated, NK cell resting, and T cell CD4+ memory resting elevated (P < 0.05) (Fig. 7G–L). In total, 45 ICGs were linked to risk signature, with the exception of TNFRSF14, VTCN1, CD276, and CD44, which were overexpressed in the high-risk group. For the rest 42 genes, which include BTLA, CD200, NRP1, LAIR1, CD244, LAG3, CD200R1, ICOS, CD40LG, CTLA4, CD48, CD28, HAVCR2, ADORA2A, TNFRSF4, KIR3DL1, CD80, LAGLS9, TNFSF14, CD160, IDO2, PDCD1LG2, TNFSF18, PDCD1, TNFRSF8, CD27, BTNL2, TNFRSF25, CD40, TNFRSF18, TNFSF15, TIGIT, CD274, TNFSF4, CD86, HHLA2, CD70, and TNFRSF9, they were associated with low-risk score (***P < 0.001, **P < 0.01, *P < 0.05) (Fig. 7M). The low-risk group consistently outperformed the high-risk group in all three TME subcategories (stromal, immune, and ESTIMATE) (***P < 0.001) (Fig. 7N). Low-risk SKCM patients showed better TIDE prediction scores than high-risk SKCM patients, but both were below 0 (***P < 0.001) (Fig. 7O).

### Association with m6A, m5C, and m7G-related genes

**Figure 8:**
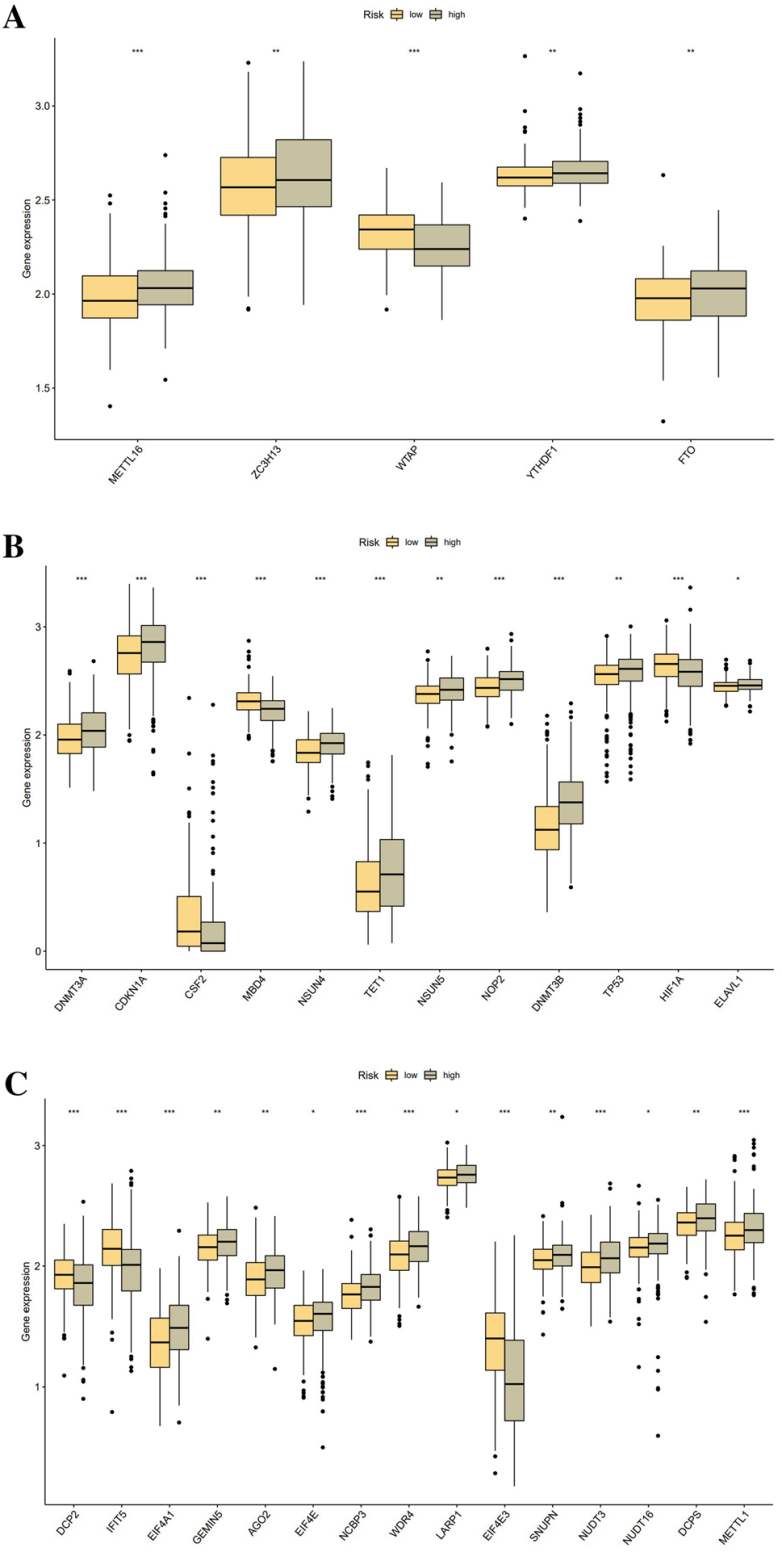
RNA methylation-related gene expression in distinct risk scores. (**A**) Differential expression of m6A genes. (**B**) Differential expression of m5C genes. (**C**) Differential expression of m7G genes.

As revealed by the findings of the differences analysis, there were substantial variations in the expression of m6A methylation-related genes, including METTL16, ZC3H13, WTAP, YTHDF1, and FTO, between risk groups. Among them, only WTAP was more expressed in the low-risk group, while the other four genes were in the opposite situation (***P < 0.001, **P < 0.01) (Fig. 8A). Altogether 12 m5C methylation-related genes were differentially expressed, with DNMT3A, CDKN1A, NSUN4, TET1, NSUN5, NOP2, DNMT3B, TP53, and ELAVL1 being more abundantly conveyed in the high-risk group, CSF2, MBD4, and HIF1A were overexpressed in the low-risk group (***P < 0.001, **P < 0.01, *P < 0.05) (Fig. 8B). Besides, approximately half of the m7G methylation-related genes were differentially expressed between risk groups. DCP2, IFIT5, and EIF4E3 were substantially upregulated in the low-risk group. However, EIF4A1, GEMIN5, AGO2, EIF4E, NCBP3, WDR4, LARP1, SNUPN, NUDT3, NUDT16, DCPS, and METTL1 were enriched in high-risk subset (***P < 0.001, **P < 0.01, *P < 0.05) (Fig. 8C).

### Drug sensitivity prediction for risk signature

**Figure 9:**
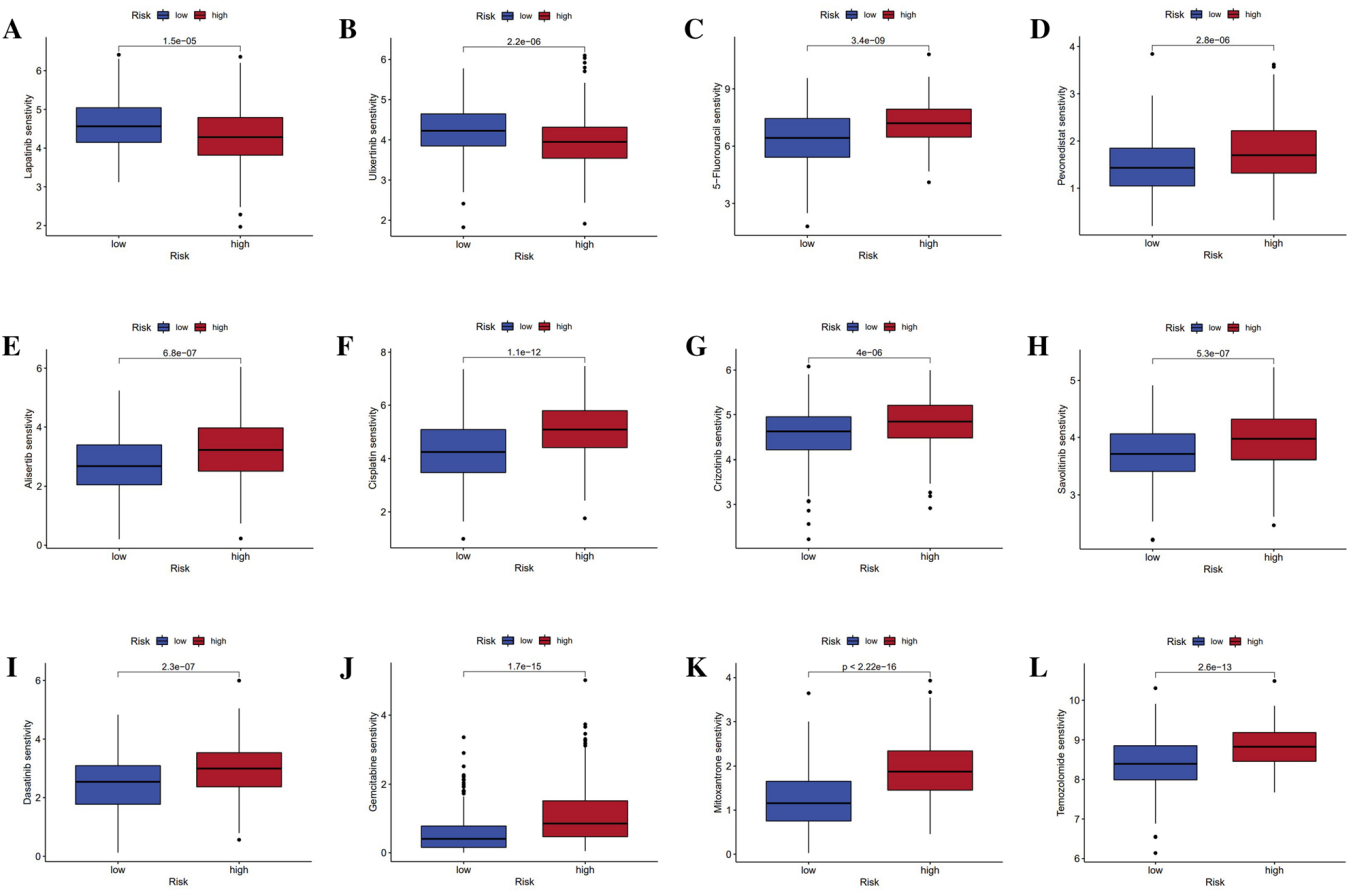
Sensitivity drug prediction for melanoma. (**A**–**L**) IC50 prediction for Lapatinib, Ulixertinib, 5-Fluorouracil, Alisertib, Cisplatin, Crizotinib, Dasatinib, Gemcitabine, Mitoxantrone, Pevonedistat, Savolitinib, and Temozolomide.

With the “oncoPredict” R package, the IC50 of many anti-cancer drugs for the risk signature was predicted. For both Lapatinib and Ulixertinib, the IC50 scores were lower in the high-risk group, indicating that these patients were more suitable for treatment (P < 0.001) (Fig. 9A,B). Nevertheless, ten commonly prescribed anti-cancer drugs exhibited lower IC50s in the low-risk subgroup, including 5-Fluorouracil, Alisertib, Cisplatin, Crizotinib, Dasatinib, Gemcitabine, Mitoxantrone, Pevonedistat, Savolitinib, and Temozolomide (P < 0.001) (Fig. 9C–L).

## Discussion

Cutaneous melanoma is a skin malignancy predisposed to metastasis. Therefore, when an early and accurate diagnosis is missed, the prognosis for progressive melanoma shows poor results^1,3^. For some end-stage patients suffering from unresectable or metastatic malignant melanoma, integrated treatment with BRAF-targeted and MEK-targeted therapies and the emergence of immunotherapy resulted in a significant improvement in OS^7,8^. But the growing occurrence of drug resistance and multiple adverse effects would be a severe challenge for the future of melanoma treatment^6,24^. Anoikis typically occurs after the cells detach from the ECM and surrounding cells, which has a potentially crucial role in tumor metastasis and angiogenesis^13^. Inducing anoikis will not only prevent tumor metastasis but also boost the sensitivity of chemotherapeutic drugs for cancer cells^15,25^. Many studies have found that lncRNAs mediate tumor metastasis in a complex and bidirectional way^26,27^. Furthermore, several lncRNAs contribute to the immune evasion of tumor cells, resulting in immunotherapy failure^21^. While some other lncRNAs may modulate the tumor microenvironment and eliminate immunotherapeutic resistance^19,20^. Therefore, identifying prognostic markers based on anoikis-related lncRNAs could provide not only novel perspectives for studying melanoma metastasis but also offer new potential therapeutic targets for anti-cancer drug resistance of advanced tumor patients.

Through this research we conducted, 4 anoikis-related prognostic lncRNAs were found, including LINC01711, MIR205HG, LINC02416, and POLH-AS1. Our study showed that the above 4 hub lncRNAs were associated with BAX, BAD, CASP8, CASP3, EGFR, AKT-, and MAPK-family genes, which were critical genes in the process of anoikis or anoikis-resistance in cells^12,13^. MIR205HG was a prognostic marker for melanoma and had a specific role in the immune and epidermal developmental pathways of the tumor^28^. EGFR, CEACAM6, CEACAM5, MAPK3, and AKT2 were identified to show a remarkable positive relationship with MIR205HG, according to our study. Existing studies show that MIR205HG could activate MAPK and PI3K/AKT signal pathways to interfere with the progression of hepatoblastoma^29^. Overexpression of MIR205HG also contributed to the malignant proliferation in head and neck squamous carcinoma cells^30^. LINC01711, LINC02416, and POLH-AS1 were first found to be associated with the OS of melanoma. From the results of the Sankey diagram and the correlation heat map, LINC01711 was found to be highly relevant to 35 ARGs. Among them, it was significantly positively correlated with TIMP1, while significantly negatively correlated with BCL2. Research has shown that inhibition of BCL2 regulates the M2 macrophage depolarization, which promotes anti-cancer immunity and interferes with tumor progression^31^. Furthermore, during our study, ITGA family genes exhibited a high positive correlation with LINC01711, and these genes were shown to be implicated in the modulation of the TME and tumor metastasis in melanoma^32,33^. LINC02416 was found to be positively relevant to CFLAR and PTK2B and conversely negative with BSG, according to our findings. The role of these genes in cutaneous melanoma is not yet known, but it may be a new therapeutic target in the near future. POLH-AS1 displayed a highly positive correlation with PTRH2. PTRH2 could affect key cellular processes involving cell reproduction and differentiation in response to adhesion by modulating the expression of Bcl2, PI3K/AKT, and ERK signaling pathways, which could promote cancer progression and metastasis^34^. However, its role in melanoma has yet to be reported in any research. These anoikis-related hub lncRNAs acquired through our study and their concrete mechanisms of intervention with many related genes need to be explored in more studies, but this may provide a research foundation for anoikis to become a potential therapy for cutaneous melanoma.

Melanoma is a highly immunogenic cancer with sensitivity to ICIs^11^. Our results demonstrated that anoikis-related lncRNAs might influence the TME of melanoma by regulating the expression of related immune molecules. The low-risk group had a greater level of B cell, Macrophage M1 cell, NK cell, and CD8+ T cell immune infiltration. CD8+ T cell was the key immune cell activated during anti-CTLA4 and anti-PD-1 immunotherapy procedures^10^. B cell, Macrophage cell, and NK cell have been proven to be essential for the malignant proliferation and polarization of tumor cells^35^. M1 macrophages could promote the generation of inflammatory factors and attain anti-tumor function^36^. On the contrary, M2 macrophages produce VEGF, TGF-β, PDGF, and other cytokines, which facilitate tumor angiogenesis and Epithelial–Mesenchymal Transition (EMT), causing immunosuppression and immune evasion in TME, resulting in tumor progression or metastasis^37,38^. These would also conform to our findings that the low-risk subgroup had a more extent infiltration of M1 macrophages and a lower M2 macrophages infiltration. And most ICGs showed markedly greater expression levels in the low-risk group. Among them, LAG3, CD28, CD80, CD86, and CTLA4 played a determinant role in immunotherapy against CTLA4 and PD-1^39,40^. By analyzing the results of TME scores for distinct risk characteristics, the immune performance was superior in the low-risk group. Stromal scores converged to 0 across both risk groups, implying a lack of cancer stem cells (CSCs) infiltration within the immune microenvironment of melanoma. CSCs have low immunogenicity and suppress the immune response, which is of particular implication in cancer invasion, recurrence, and therapy resistance. CSCs had been increasingly studied for their weaker immunogenicity and suppression of immune responses, with particular relevance in cancer invasion, recurrence, and therapy resistance. Targeted therapy against CSCs may be a potential new modality to supplement other anti-cancer therapies^41,42^. The result of TIDE indicated a shortage of expression of the immune evasion phenotype in both risk groups. Moreover, poor response to immunotherapy in the high-risk group was consistent with the significance of the TME score results. Nevertheless, the detailed mechanisms by which anoikis-related signature lncRNAs engage in the TME of melanoma should be examined further.

RNA methylation as a mode of epigenetic regulation of tumors interfered in both mRNAs and lncRNAs. Dysregulation of RNA methylation may cause tumor progression and metastasis, impair immune cell function, and affect the immune microenvironment of tumors, rendering immunotherapy resistance or even immunotherapy failure^43,44^. Throughout the current study, N6-methyladenosine (m6A) remains the most typically explored RNA methylation variation, which primarily impacts RNA metabolism. In our study, the higher risk score was associated with a lower expression of WTAP and a higher expression of METTL16, ZC3H13, YTHDF1, and FTO. It has been demonstrated that METTL16 catalyzed m6a modification, ZC3H13, and WTAP combined to participate in the composition of m6a methyltransferase, YTHDF1 promoted the translation of m6A-binding proteins, and FTO-activated demethylases to eliminate m6A modification^45,46^. DNMTs and NSUNs had been shown in existing studies to participate in m5C writers, which induce the migration and metastasis of tumor cells^47,48^. Box plots obtained from our study showed that DNMT3A, DNMT3B, NSUN4, NSUN5, and NOP2 were all positively related to risk scores. Similarly, METTL1 and WDR4 were found to be substantially upregulated in the high-risk group, and these two genes have been identified as one of the primary regulatory axes involved in m7G methylation^49,50^. The outcomes of this study revealed that samples with high-risk scores exhibited a high degree of RNA methylation, which probably contributed to the poorer immunological status of the high-risk group. However, the exact mechanism by which RNA methylation cooperates with anoikis-related lncRNAs to modify the TME is yet to be revealed.

With the development of pharmacotherapy for advanced melanoma, treatment options of targeted drugs in conjunction with ICIs are gradually becoming beneficial in clinical practice^8,9^. But the lack of highly specific and sensitive prognostic markers contributes to the fact that there are still patients with an inadequate response to immunotherapy and many adverse events, leading to poor outcomes^6,10,11,39^. In the present study, a total of 12 anti-cancer drugs with favorable sensitivity were filtered, and only two drugs were more strongly correlated with high-risk scores. Lapatinib and Dasatinib are representative drugs of receptor tyrosine kinase (RTK) inhibitors, whose combination with nelfinavir may prevent drug resistance in melanoma^51^. Ulixertinib and Pevonedistat may synergize with BRAF-targeted inhibitors to produce an anti-malignant proliferation of melanoma cells^52–54^. Traditional chemotherapeutics, including Temozolomide and Cisplatin, have been shown to trigger an immune response and enhance the responses to immunotherapy in melanoma^55,56^. Crizotinib may be used as a complementary therapy for melanoma with BRAF mutations by inhibiting the mTOR/Insulin signaling pathway^57,58^. Therefore, these hypersensitive anti-cancer drugs would be synergistic with targeted inhibitors and immunotherapy for melanoma.

## Conclusion

In the current view of our study, the successful acquisition of prognostic signatures based on four anoikis-related lncRNAs has important clinical meanings and offers potentially novel therapeutic targets for eliminating resistance to BRAF-targeted inhibitors, MEK-targeted inhibitors, and immunotherapy in cutaneous melanoma, but limitations remain. Firstly, there is still a lack of external validation of additional databases and experimental analysis to back up the results of our study. Secondly, more research needs to be conducted on the mechanisms by which these four lncRNAs modulate mRNA expression and RNA methylation to interfere with the TME and affect the immunotherapeutic response to cancer. Moreover, it’s necessary to have clinical efficacy data from multi-center trials and prospective studies to demonstrate the applicability of the drug sensitivity results.

### Supplementary Information

Supplementary Tables.

## Data Availability

The datasets used for analysis in this study were drawn from open access databases, including the TCGA (https://portal.gdc.cancer.gov/), the GTEx database (https://xenabrowser.net/), and the GDSC database (https://www.cancerrxgene.org/).
