# Supplementary material for: A novel signature for predicting prognosis and immune landscape in cutaneous melanoma based on anoikis-related long non-coding RNAs
Source: Sci Rep. 2023 Sep 28;13:16332. doi: 10.1038/s41598-023-39837-5 (PMC10539372; doi:10.1038/s41598-023-39837-5)
Supplement: Supplementary file 1 — Supplementary Tables. [file 41598_2023_39837_MOESM1_ESM.doc]

***Supplementary Material***

**A novel signature for predicting prognosis and immune landscape in cutaneous melanoma: based on anoikis-related long non-coding RNAs**

**Miao Zhang^1^, Yuzhi Zuo^1^,** **Jian Guo^2^, Lushan Yang^1^, Yizhi Wang^1^, Meiyun Tan**^3^***，Xing Guo^1,4^***

*** Correspondence:** Meiyun Tan, drtmy169@swmu.edu.cn; Xing Guo, gx412@126.com

# 1 Supplementary Tables

Table Ⅰ: Anoikis-related genes (ARGs)

| Title | Total | Relevance score > 2 |
| --- | --- | --- |
| ARGS | 67 | | BRMS1 | PTK2 | NTRK2 | BCL2L11 | SRC | | --- | --- | --- | --- | --- | | CEACAM6 | CAV1 | AKT1 | ITGB1 | CEACAM5 | | EGFR | BCL2 | CASP8 | SIK1 | PTRH2 | | STAT3 | TLE1 | DAPK2 | CTNNB1 | ZNF304 | | MAPK1 | BMF | ITGA5 | TP53 | MCL1 | | BCL2L1 | CASP3 | CDH1 | BAD | PIK3CA | | PAK1 | ITGAV | FN1 | MAPK3 | PTGS2 | | BAX | BCAR1 | PTEN | ERBB2 | ANGPTL4 | | PDK4 | CYCS | BRAF | YAP1 | ANKRD13C | | ITGA2 | ANXA5 | BIRC5 | MTOR | TIMP1 | | BDNF | CSPG4 | BSG | AKT2 | STK11 | | IGF1 | IGF1R | ITGA6 | ILK | CFLAR | | RHOA | HIF1A | DAP3 | MYBBP1A | TLE5 | | ITGA3 | PTK2B |  |  |  | |

Table Ⅱ: Immune checkpoint genes (ICGs)

| Title | Total |  |
| --- | --- | --- |
| ICGs | 45 | | IDO1 | LAG3 | CTLA4 | TNFRSF9 | ICOS | | --- | --- | --- | --- | --- | | CD80 | PDCD1LG2 | TIGIT | CD70 | TNFSF9 | | ICOSLG | KIR3DL1 | CD86 | PDCD1 | LAIR1 | | TNFRSF8 | TNFSF15 | TNFRSF14 | IDO2 | CD276 | | CD40 | TNFRSF4 | TNFSF14 | HHLA2 | CD244 | | CD274 | HAVCR2 | CD27 | BTLA | LGALS9 | | TMIGD2 | CD28 | CD48 | TNFRSF25 | CD40LG | | ADORA2A | VTCN1 | CD160 | CD44 | TNFSF18 | | TNFRSF18 | BTNL2 | NRP1 | CD200 | TNFSF4 | |

Table Ⅲ: RNA methylation-related genes

| Title | m6A | m5C | m7G |
| --- | --- | --- | --- |
|  | 19 | 23 | 29 |
| Gene | | METTL3 | METTL14 | METTL16 | | --- | --- | --- | | WTAP | KIAA1429 | RBM15 | | ZC3H13 | YTHDC1 | YTHDC2 | | YTHDF1 | YTHDF2 | YTHDF3 | | HNRNPC | HNRNPA2B1 | FTO | | ALKBH5 | IGF2BP1 | IGF2BP2 | | IGF2BP3 |  |  | | | DNMT1 | MBD4 | NSUN2 | | --- | --- | --- | | DNMT3A | DNMT3B | TP53 | | FMR1 | METTL3 | TET1 | | YBX1 | CDKN1A | TRDMT1 | | NOP2 | NSUN3 | ALYREF | | METTL14 | NSUN4 | NSUN5 | | HIF1A | ELAVL1 | NSUN6 | | YTHDF2 | CSF2 |  | | | METTL1 | WDR4 | NSUN2 | | --- | --- | --- | | DCP2 | DCPS | NUDT10 | | NUDT11 | NUDT16 | NUDT3 | | NUDT4 | NUDT4B | AGO2 | | CYFIP1 | EIF4E | EIF4E1B | | EIF4E2 | EIF4E3 | GEMIN5 | | LARP1 | NCBP1 | NCBP2 | | NCBP3 | EIF3D | EIF4A1 | | EIF4G3 | IFIT5 | LSM1 | | NCBP2L | SNUPN |  | |

Table Ⅳ: Differentially expressed lncRNAs

| DELs | logFC | pValue | FDR | DELs | logFC | pValue | FDR |
| --- | --- | --- | --- | --- | --- | --- | --- |
| ENTPD1-AS1 | -2.7203 | 8.05E-194 | 9.50E-192 | LINC02446 | 3.890447 | 1.30E-111 | 4.46E-111 |
| LINC00592 | -2.73634 | 8.41E-113 | 2.92E-112 | LINC01857 | 6.119175 | 3.27E-215 | 7.73E-213 |
| USP30-AS1 | 2.760773 | 7.71E-114 | 2.76E-113 | IDI2-AS1 | 2.213187 | 1.75E-133 | 8.25E-133 |
| LINC01711 | 3.060978 | 4.16E-97 | 1.28E-96 | LINC02560 | -3.00375 | 1.57E-85 | 4.11E-85 |
| LINC01215 | 3.727769 | 8.67E-66 | 1.61E-65 | LINC02437 | -3.43739 | 7.24E-75 | 1.63E-74 |
| PIK3CD-AS2 | 2.666128 | 3.80E-190 | 1.24E-188 | LINC00528 | 2.096196 | 4.25E-66 | 8.09E-66 |
| LINC01836 | -2.82217 | 7.41E-163 | 6.72E-162 | LINC01348 | -3.61256 | 3.72E-152 | 2.44E-151 |
| MLIP-IT1 | -3.62323 | 5.02E-72 | 1.07E-71 | LINC01527 | -2.29657 | 7.57E-69 | 1.53E-68 |
| LINC02273 | 2.405995 | 2.20E-54 | 3.47E-54 | POLH-AS1 | 2.244899 | 2.01E-178 | 3.64E-177 |
| LINC01550 | -3.48083 | 4.23E-160 | 3.32E-159 | FAM30A | 2.961288 | 1.37E-66 | 2.63E-66 |
| LINC00518 | 4.328108 | 4.09E-178 | 6.90E-177 | EMX2OS | -3.47479 | 7.63E-192 | 4.50E-190 |
| MIR3142HG | 3.414802 | 1.60E-187 | 4.21E-186 | LINC02178 | -3.09304 | 6.58E-67 | 1.28E-66 |
| FIRRE | 3.313589 | 2.87E-148 | 1.78E-147 | SEMA6A-AS2 | 3.647092 | 1.56E-146 | 8.99E-146 |
| LINC01781 | 4.487895 | 4.95E-94 | 1.43E-93 | PLA2G4E-AS1 | -4.15462 | 3.26E-46 | 4.72E-46 |
| IRAIN | 2.356234 | 6.25E-95 | 1.82E-94 | STK32A-AS1 | 4.057376 | 1.80E-171 | 2.36E-170 |
| LINC00885 | -3.40359 | 1.14E-56 | 1.90E-56 | LINC00582 | 2.389117 | 3.67E-36 | 4.95E-36 |
| TRBV11-2 | 4.747191 | 1.87E-147 | 1.13E-146 | LINC02084 | 3.112705 | 1.06E-155 | 7.59E-155 |
| MIR200CHG | -3.33158 | 1.07E-86 | 2.88E-86 | LURAP1L-AS1 | 2.216348 | 3.25E-56 | 5.36E-56 |
| LINC01443 | 4.325295 | 3.53E-98 | 1.10E-97 | CADM3-AS1 | -3.19558 | 8.21E-168 | 9.68E-167 |
| RHPN1-AS1 | 3.42903 | 1.97E-192 | 1.55E-190 | LINC02528 | 5.784859 | 5.84E-142 | 2.93E-141 |
| SOX21-AS1 | -3.23762 | 8.12E-71 | 1.67E-70 | IER3-AS1 | 2.442178 | 1.23E-167 | 1.38E-166 |
| FZD10-AS1 | -3.57315 | 6.71E-84 | 1.68E-83 | SUCLA2-AS1 | 3.41553 | 1.50E-191 | 7.09E-190 |
| BASP1-AS1 | 2.699078 | 6.72E-37 | 9.17E-37 | LINC01805 | -4.7386 | 2.36E-82 | 5.75E-82 |
| LINC02422 | 3.597451 | 9.02E-81 | 2.17E-80 | LINC01133 | -3.86767 | 4.19E-190 | 1.24E-188 |
| CALML3-AS1 | -4.49385 | 2.68E-80 | 6.39E-80 | CHRM3-AS2 | 2.248241 | 6.43E-22 | 7.67E-22 |
| UCA1 | -2.60156 | 5.39E-95 | 1.59E-94 | LINC00996 | 2.967642 | 6.62E-106 | 2.14E-105 |
| LINC00861 | 2.839116 | 6.05E-66 | 1.14E-65 | C8orf34-AS1 | -3.00437 | 8.84E-87 | 2.43E-86 |
| LHFPL3-AS1 | 4.471472 | 1.59E-150 | 1.02E-149 | LINC02576 | 3.876217 | 1.12E-121 | 4.25E-121 |
| LINC01621 | -2.28996 | 1.27E-54 | 2.02E-54 | MIR205HG | -3.59346 | 7.86E-147 | 4.64E-146 |
| LINC02285 | 3.863615 | 1.26E-155 | 8.76E-155 | LINC02416 | 6.990393 | 6.42E-168 | 7.98E-167 |
